# Supplementary material for: Pervasive context-dependent effects in the genetic architecture of complex and quantitative traits revealed by a powerful multiparent mapping population in yeast
Source: bioRxiv. 2025 Oct 27:2025.10.27.683165. Preprint. [Version 1] doi: 10.1101/2025.10.27.683165 (PMC12636336; doi:10.1101/2025.10.27.683165)
Supplement: Supplement 1 [file NIHPP2025.10.27.683165v1-supplement-1.pdf]

bioRxiv preprint doi: https://doi.org/10.1101/2025.10.27.683165; this version posted October 27, 2025. The copyright holder for this preprint (which was not certified by peer review) is the author/funder, who has granted bioRxiv a license to display the preprint in perpetuity. It is made available under aCC-BY 4.0 International license.

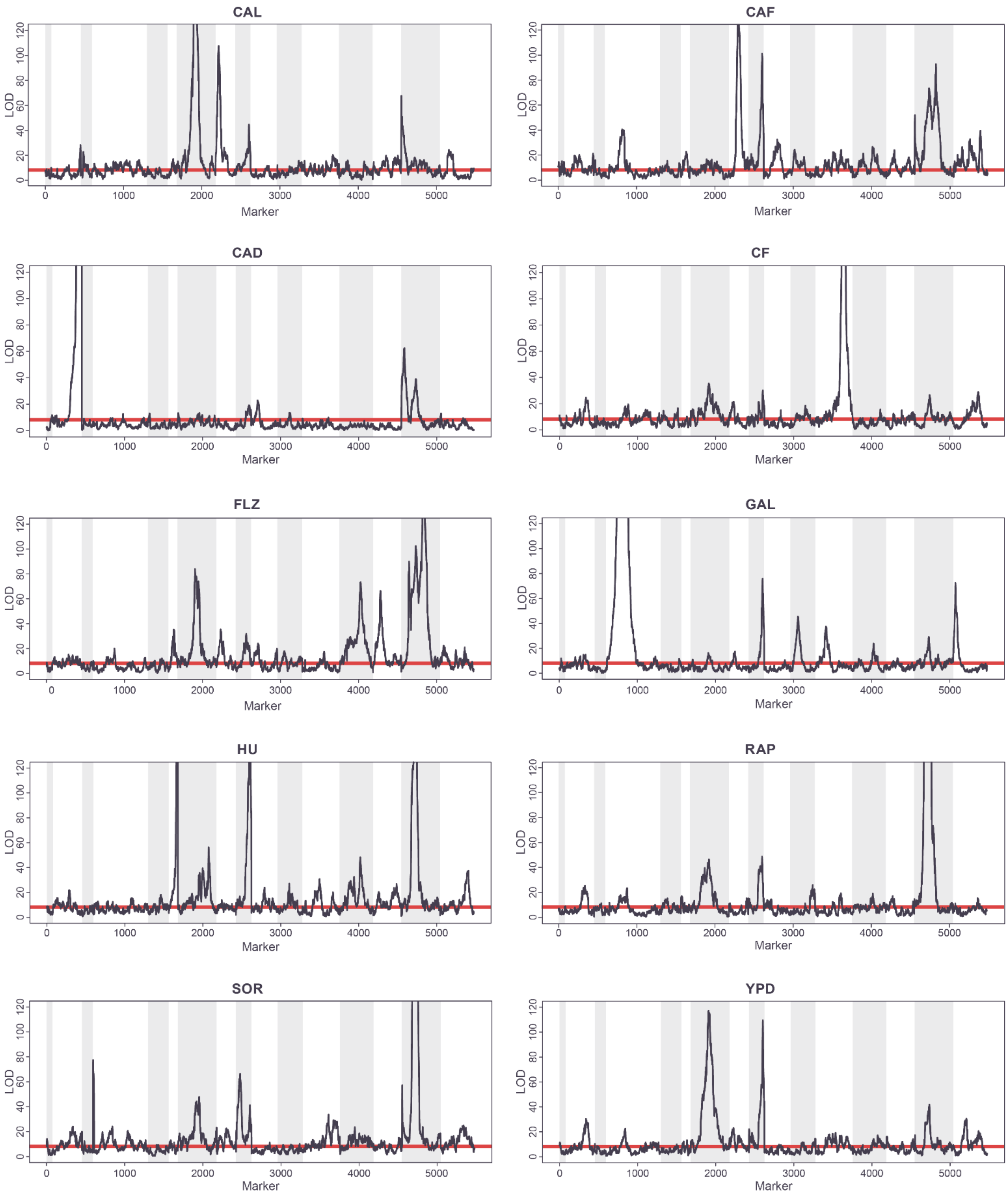

Fig S1. LOD traces for all conditions at 72h. Scaffolding marker set used. Chromosomes indicated by alternating grey and white backgrounds. Red line represents 5% family-wise significance threshold.

bioRxiv preprint doi: https://doi.org/10.1101/2025.10.27.683165; this version posted October 27, 2025. The copyright holder for this preprint (which was not certified by peer review) is the author/funder, who has granted bioRxiv a license to display the preprint in perpetuity. It is made available under aCC-BY 4.0 International license.

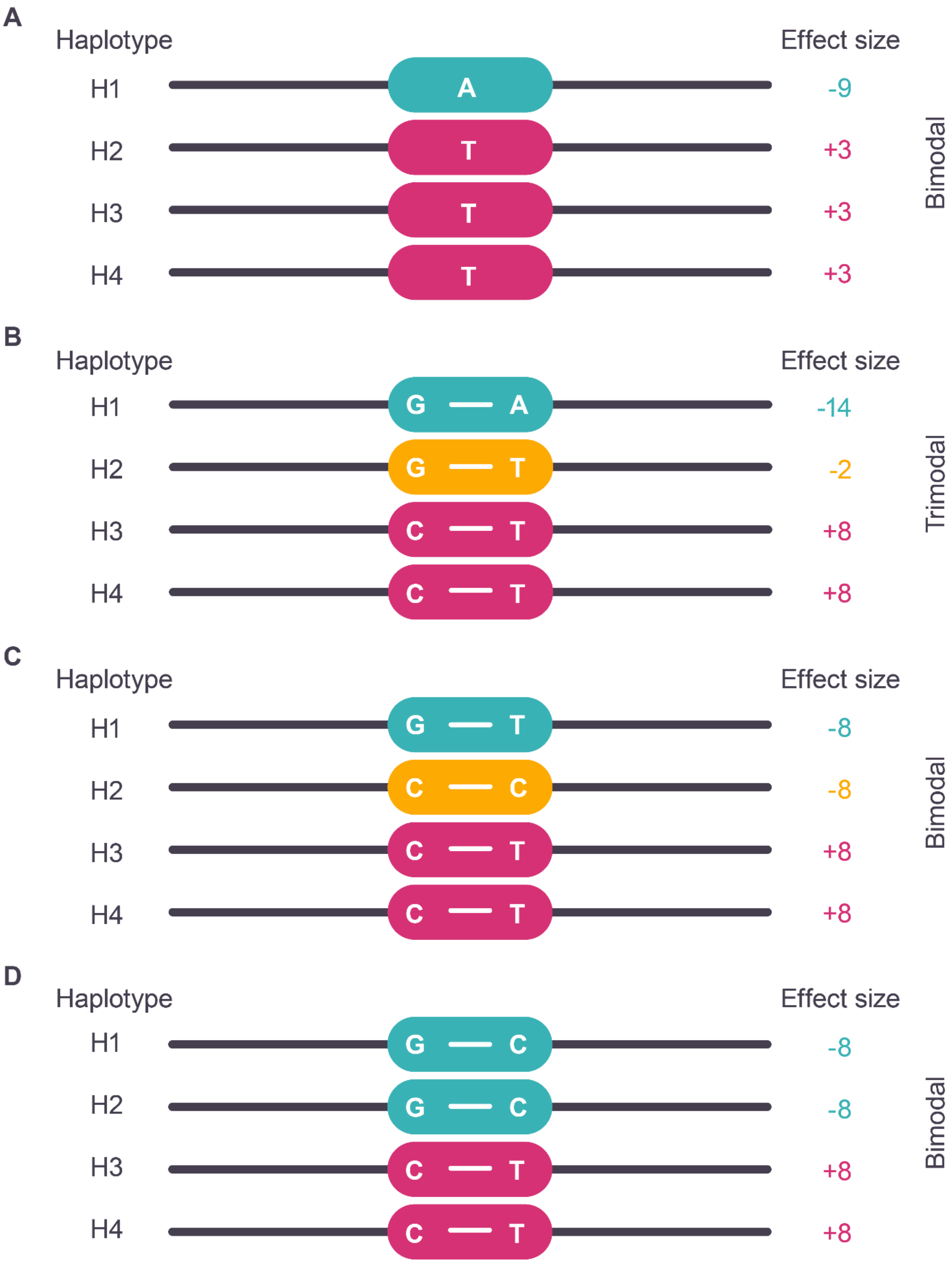

Fig S2. Patterns of haplotype effect sizes at loci with single causative variants or closely linked pairs of causative variants. (**A**) A single causative variant site always produces a bimodal effect distribution. (**B**) Two closely linked causative variant sites permuted among founder haplotypes can produce a polymodal haplotype effect distribution (e.g. G=-2, C=+8, A=-12, T=0). (**C**) Two closely linked causative variant sites permuted among founder haplotypes can produce a bimodal haplotype effect distribution if multiple allele permutations share the same effect size. (**D**) Two closely linked variant sites that are not permuted among founder haplotypes produce a bimodal effect distribution.

bioRxiv preprint doi: https://doi.org/10.1101/2025.10.27.683165; this version posted October 27, 2025. The copyright holder for this preprint (which was not certified by peer review) is the author/funder, who has granted bioRxiv a license to display the preprint in perpetuity. It is made available under aCC-BY 4.0 International license.

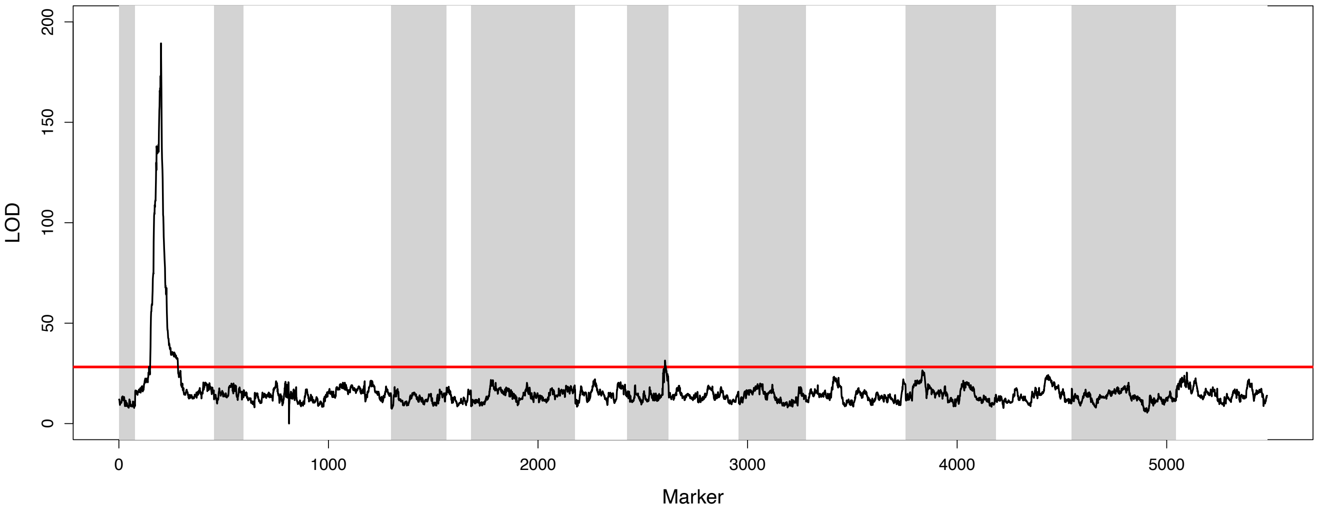

Fig S3. Interaction vs additive LOD trace for *GAL3* maximum marker (chr04_465458) vs all scaffolding markers identifies a strong interaction between *GAL3* and *GAL1/7/10*. Chromosomes indicated by alternating grey and white backgrounds. Red line represents 1% FDR threshold for full GAL interaction vs additive dataset.

bioRxiv preprint doi: https://doi.org/10.1101/2025.10.27.683165; this version posted October 27, 2025. The copyright holder for this preprint (which was not certified by peer review) is the author/funder, who has granted bioRxiv a license to display the preprint in perpetuity. It is made available under aCC-BY 4.0 International license.

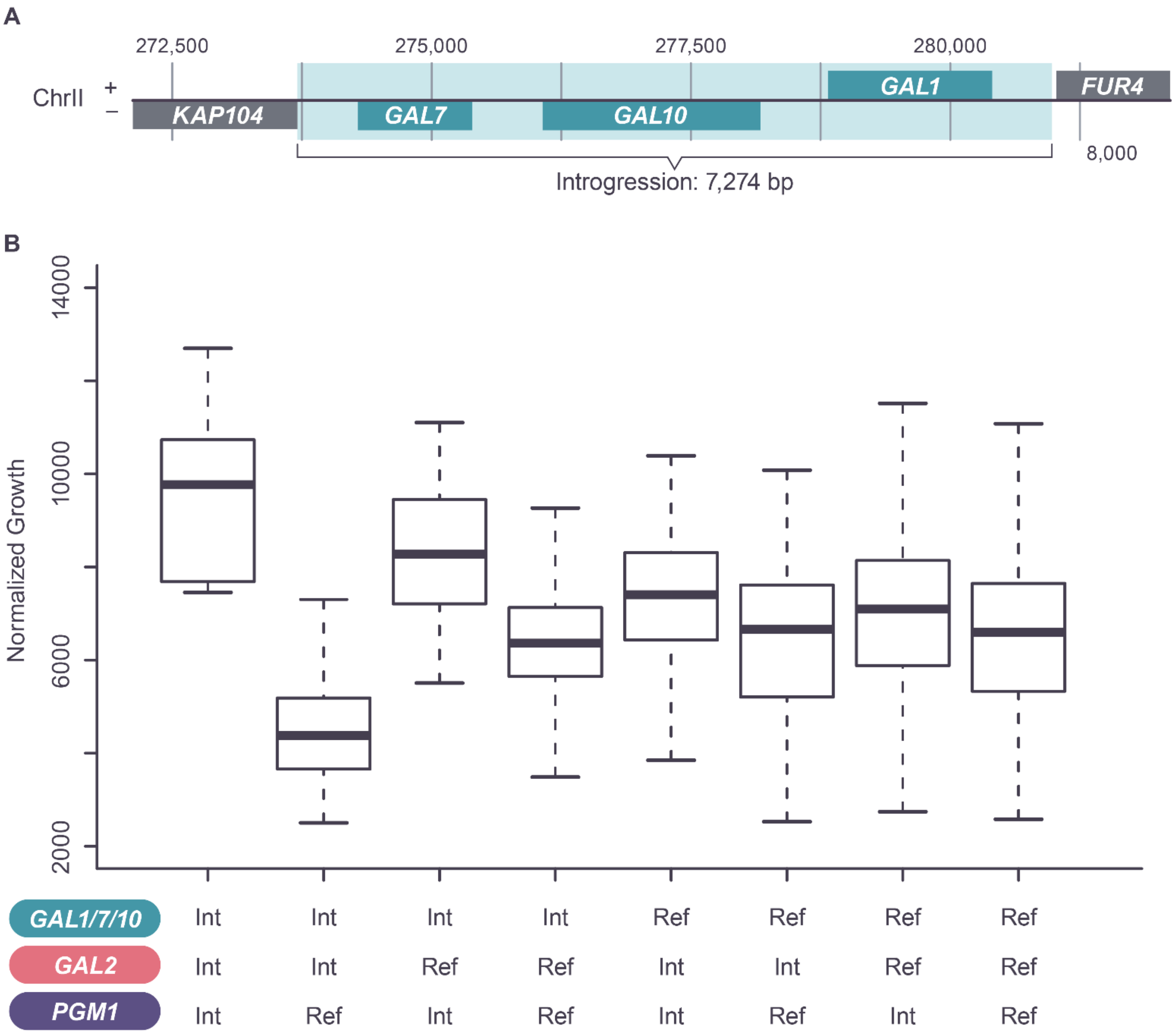

Fig S4. Interactions between reference and introgressed GAL pathway genes. (**A**) An introgression replaces the *GAL7*, *GAL10* and *GAL1* genes with non-native versions in founder 6. (**B**) Effect on strain growth of combining introgressed and non-introgressed (reference) alleles of GAL pathway genes.

bioRxiv preprint doi: https://doi.org/10.1101/2025.10.27.683165; this version posted October 27, 2025. The copyright holder for this preprint (which was not certified by peer review) is the author/funder, who has granted bioRxiv a license to display the preprint in perpetuity. It is made available under aCC-BY 4.0 International license.

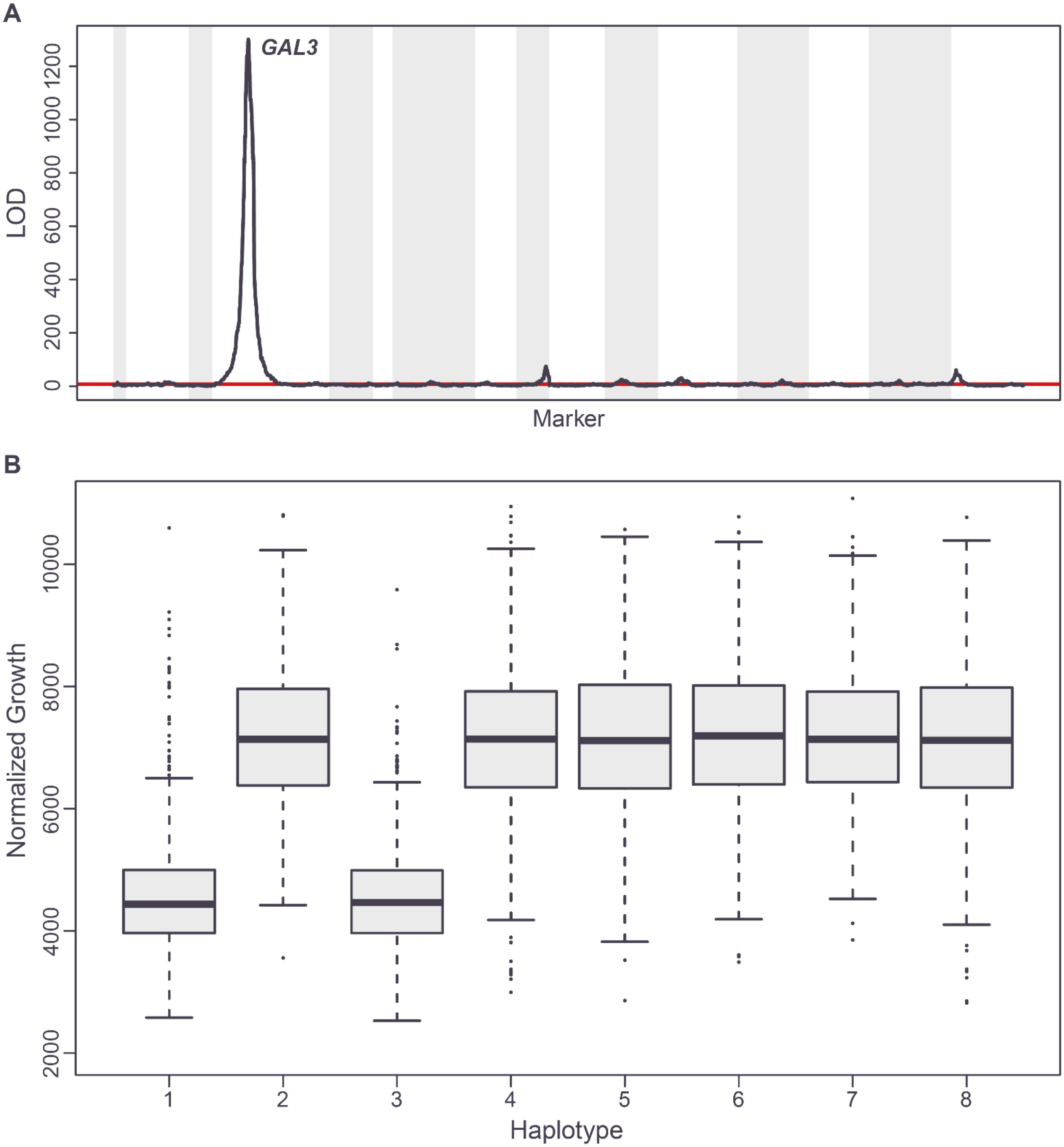

Fig S5. Effect of *GAL3* locus on growth on galactose for strains lacking the *GAL1/7/10* introgression. (**A**) LOD scores for linkage mapping on galactose. Chromosomes alternating in grey and white. 1% significance threshold in red. (**B**) Growth on galactose for strain subpopulations defined by *GAL3* haplotype.

bioRxiv preprint doi: https://doi.org/10.1101/2025.10.27.683165; this version posted October 27, 2025. The copyright holder for this preprint (which was not certified by peer review) is the author/funder, who has granted bioRxiv a license to display the preprint in perpetuity. It is made available under aCC-BY 4.0 International license.

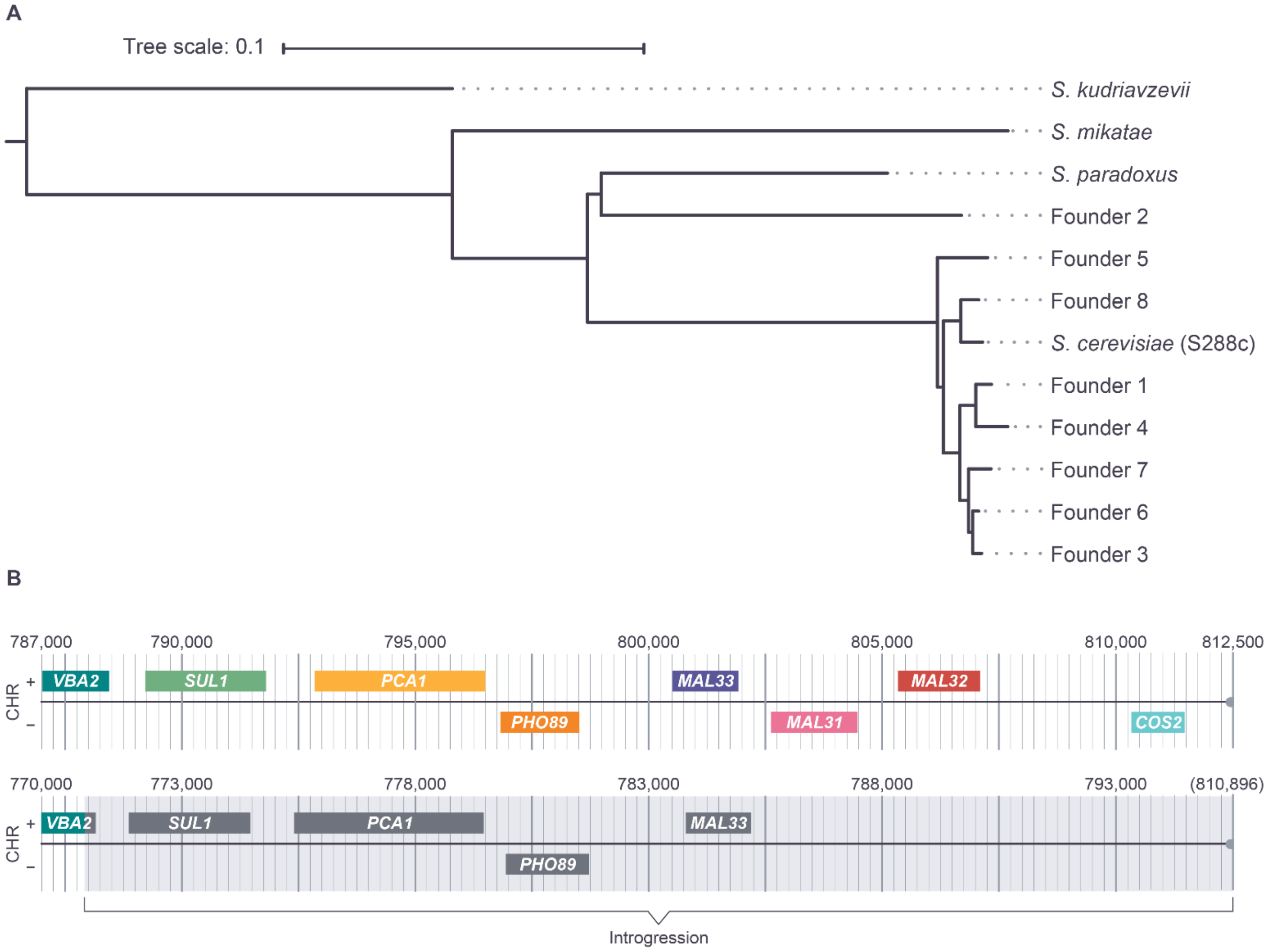

Fig S6. Founder 2 possesses an introgression on the right telomeric region of chromosome II that includes *PCA1*. (**A**) Neighbor joining tree of *PCA1* protein sequences. (**B**) The introgressed telomeric region of chromosome II from founder 2 (numbering from File S1) compared to the S288c reference. All ORFs >500bp displayed, with reference homolog names.

bioRxiv preprint doi: https://doi.org/10.1101/2025.10.27.683165; this version posted October 27, 2025. The copyright holder for this preprint (which was not certified by peer review) is the author/funder, who has granted bioRxiv a license to display the preprint in perpetuity. It is made available under aCC-BY 4.0 International license.

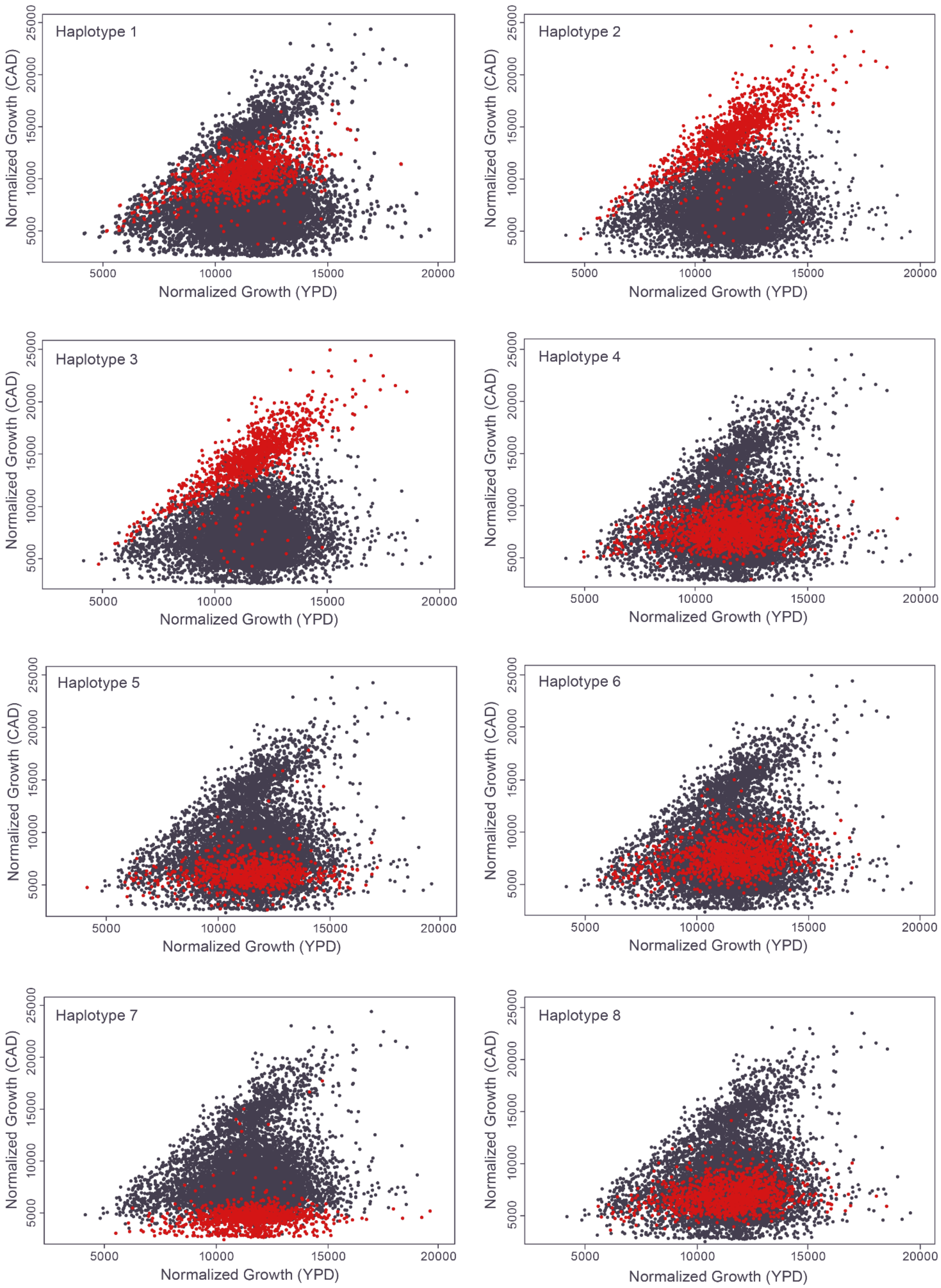

Fig S7. Comparisons between growth on YPD and CAD for strains stratified by *PCA1* haplotype. Full distribution of all strains shown in each plot, with current subset of strains indicated in red.

bioRxiv preprint doi: https://doi.org/10.1101/2025.10.27.683165; this version posted October 27, 2025. The copyright holder for this preprint (which was not certified by peer review) is the author/funder, who has granted bioRxiv a license to display the preprint in perpetuity. It is made available under aCC-BY 4.0 International license.

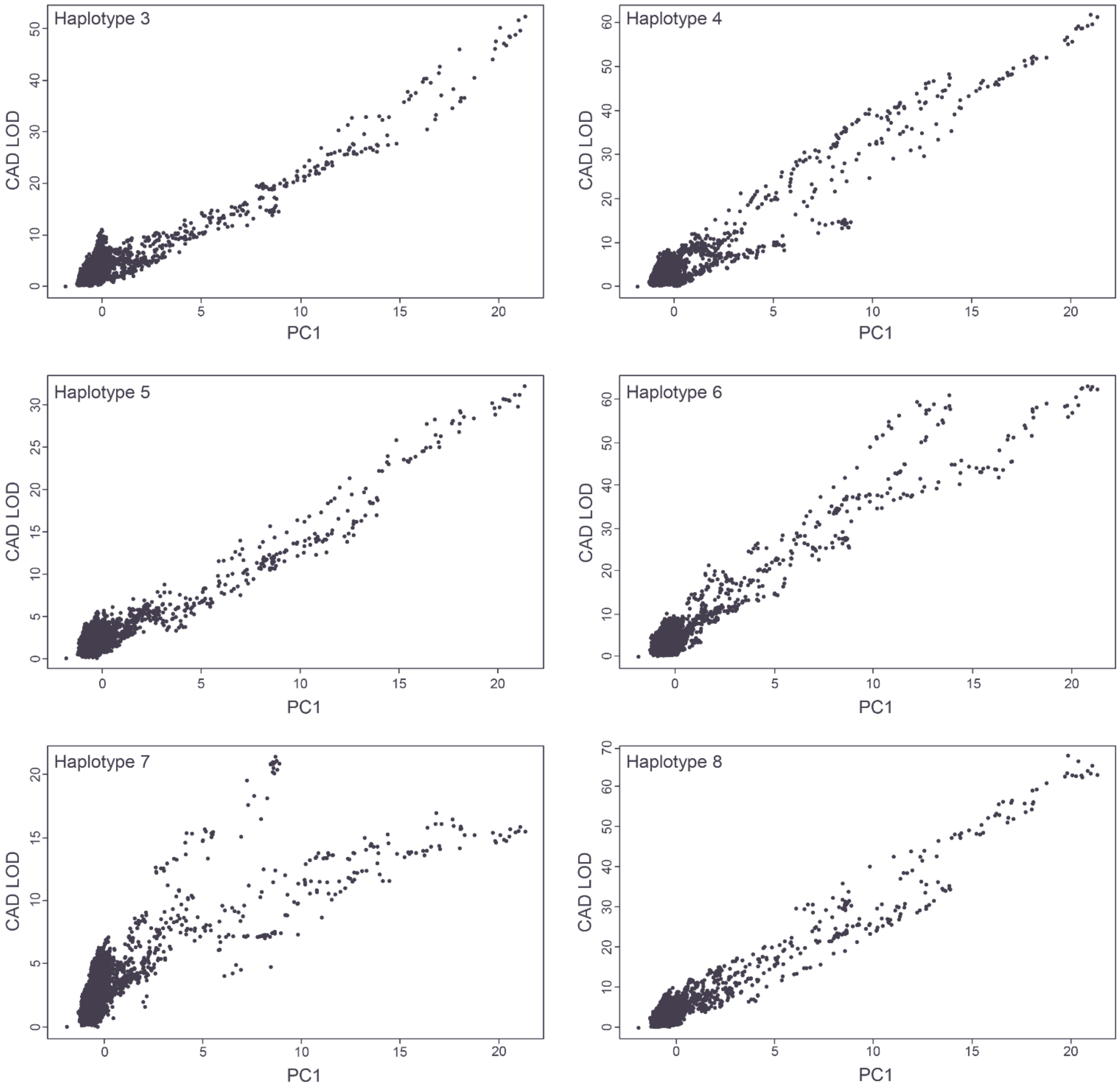

Fig S8. Shared Genetic Architecture on CAD for strains with *PCA1* haplotypes 3-8. LOD scores calculated at each scaffolding marker in each subpopulation plotted against first principal component of the subpopulation LODs (each scaled to unit variance).
